# Mapping landscape ecological patterns using numeric and categorical maps

**DOI:** 10.1371/journal.pone.0291697

**Published:** 2023-11-15

**Authors:** Kurt Riitters, Peter Vogt

**Affiliations:** 1 United States Department of Agriculture, Forest Service, Research Triangle Park, North Carolina, United States of America; 2 European Commission, Joint Research Centre, Ispra, Italy; Zoological Survey of India, INDIA

## Abstract

The reciprocal relationships between ecological process and landscape pattern are fundamental to landscape ecology. Landscape ecologists traditionally use raster maps portraying classified features such as land use or land cover categories, and metrics suggested by the patch-corridor-matrix conceptual model of pattern. Less attention has been given to the landscape gradient conceptual model and raster maps portraying numeric features such as greenness or percent vegetation cover. We introduce the open-source tool *GraySpatCon* to calculate and map a variety of landscape pattern metrics from both conceptual models using either categorical or numeric maps. The 51 metrics, drawn mostly from the landscape ecology and image processing literatures, are calculated from the frequencies of input pixel values and/or the pixel value adjacencies in an analysis region. *GraySpatCon* conducts either a moving window analysis which produces a continuous map of a pattern metric, or a global analysis which produces a single metric value. We describe an implementation in the GuidosToolbox desktop application which allows novice users to interactively explore *GraySpatCon* functionality. In the R desktop environment, we demonstrate several metrics using an example map of percent tree cover and illustrate a multi-scale moving window analysis to identify scale domains. Comparisons of computational efficiency indicate a substantial *GraySpatCon* advantage over related software in the R environment.

## Introduction

The reciprocal relationships between ecological process and landscape pattern are fundamental to landscape ecology [1–3]. Landscape pattern metrics quantify the composition and configuration of the elements comprising a landscape [4, 5]. There is a long history of using quantitative and qualitative metrics to examine pattern-process relationships from various perspectives [6–8]. While process-specific ecological interpretation of individual metrics often depends upon local circumstances [c.f. 9, 10], some less-specific yet integrative metrics are commonly applied in the context of biodiversity conservation at international scale [11–13]. Recent reviews demonstrate that research is needed to improve the science and practice of landscape pattern measurement [14–16].

The quantitative analysis of landscape patterns has co-evolved with perspectives about which patterns are important to know about, in turn driven partly by the data and software available to operationalize those perspectives [17]. Early perspectives were dominated by the patch-corridor-matrix conceptual model [18] and spawned a generation of software for analysis of categorical maps [4] as exemplified by *FRAGSTATS* [19] and *landscapemetrics* [20]. The advent of maps portraying numeric data (e.g., surface maps of greenness or percent vegetation cover) has contributed to the growing popularity of an alternate conceptual model known as the landscape gradient [21] and recognition that a new generation of pattern analysis is required [22].

While some early landscape ecology software provided limited analysis of numeric data [23], recent ecological research with numeric data has typically used proprietary software from the fields of surface metrology [22, 24–26] and image processing [27–32]. Among the open-source packages available for the R desktop environment, patch-corridor-matrix metrics are available in *landscapemetrics* [20], surface metrology metrics are available in *GEODIV* [33], and image texture metrics are available in *glcm* [34] and *fastGLCM* [35]. However, none of those tools were developed for both categorical and numeric input data. Furthermore, integrated software systems designed for remote sensing (e.g., *Earth Engine* [36]), medical imaging (e.g., *3D Slicer* [37]), or geographic information systems (e.g., *GRASS* [38] and *QGIS* [39]) may provide some relevant procedures but may also be less accessible to non-specialists in those fields.

We developed the open-source C program *GraySpatCon* (Gray-scale Spatial Convolution, version 1.1.1) to support analysis of a wide range of landscape pattern metrics used by landscape ecologists, including metrics applicable to categorical or numeric raster input data. Despite the popularity of the patch-corridor-matrix conceptual model, its focus on metrics which describe discrete patches in fixed-area landscapes is inconsistent with the landscape gradient conceptual model, in which landscape pattern is a spatially continuous property of a landscape. Furthermore, in contrast to patch-level metrics, pixel-level measures are arguably the fundamental metrics of landscape pattern because patch-level metrics can often be estimated from pixel-level measures [40]. The overall objective of our project is to stimulate and facilitate the application of landscape gradient data in ecological research. The objectives of this paper are to describe the functionality of *GraySpatCon* and its implementation within several popular computing environments, to illustrate a multi-scale analysis using a selected landscape gradient metric, and to compare computational efficiency with several software alternatives.

## Methods

The 51 *GraySpatCon* metrics are calculated from frequencies of input pixel values and/or pixel value adjacencies, and *GraySpatCon* is designed primarily for moving window analyses which produce continuous maps of pattern metrics. The moving window algorithm moves an analysis window across the input map, one pixel at a time, accumulating and discarding information along the way. Based on the pixel values and/or adjacencies in the window at a given pixel location, a metric is calculated and assigned to that location on the output map. Thus, the output pixel value codes the landscape pattern context of that pixel location, and the spatial resolution of the input map is preserved. The spatial scale of a moving window analysis is defined by the size of the window; *GraySpatCon* optionally calculates metrics for a window defined as the entire map extent.

**Fig 1:**
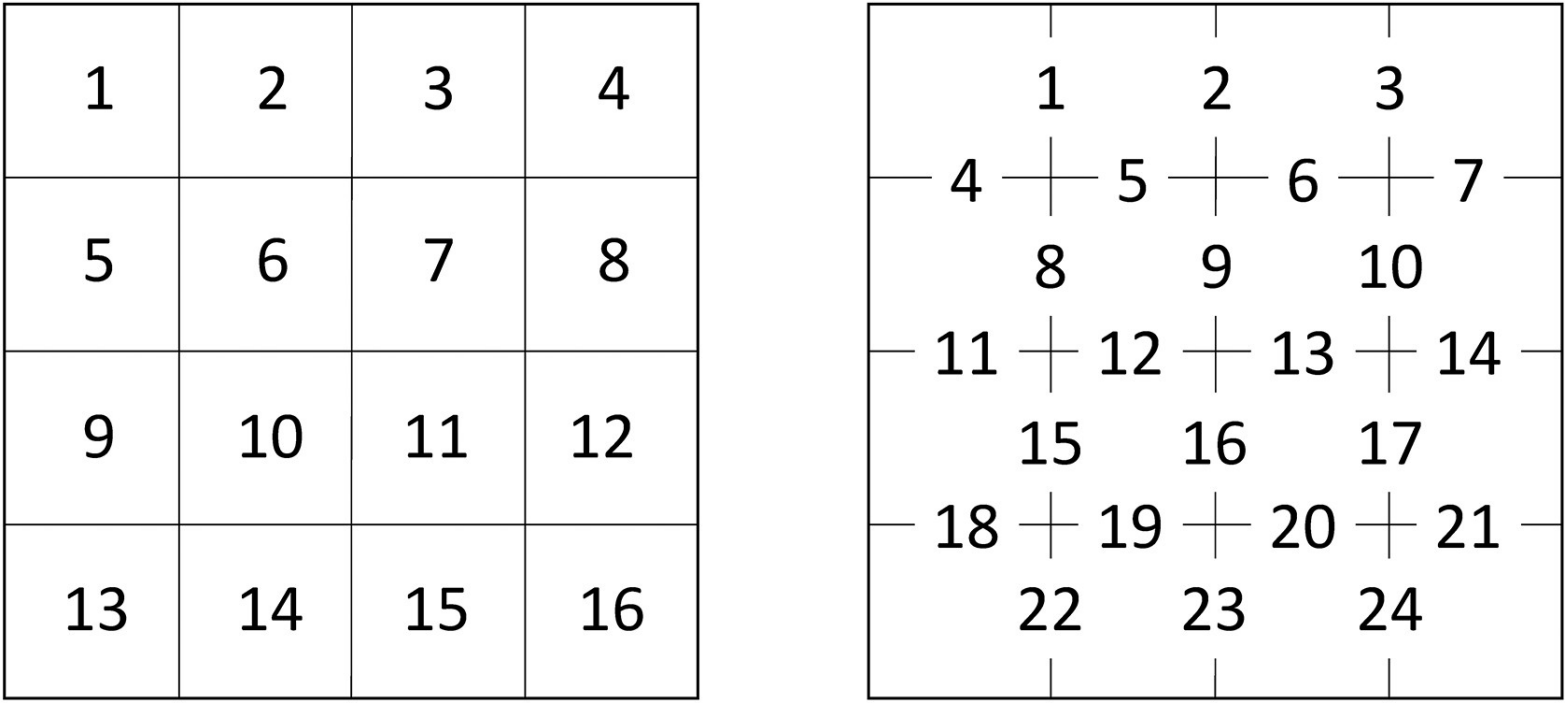
Example of a 4 pixel x 4 pixel map containing 16 pixels (left) and 24 adjacencies (right).

**Fig 2:**
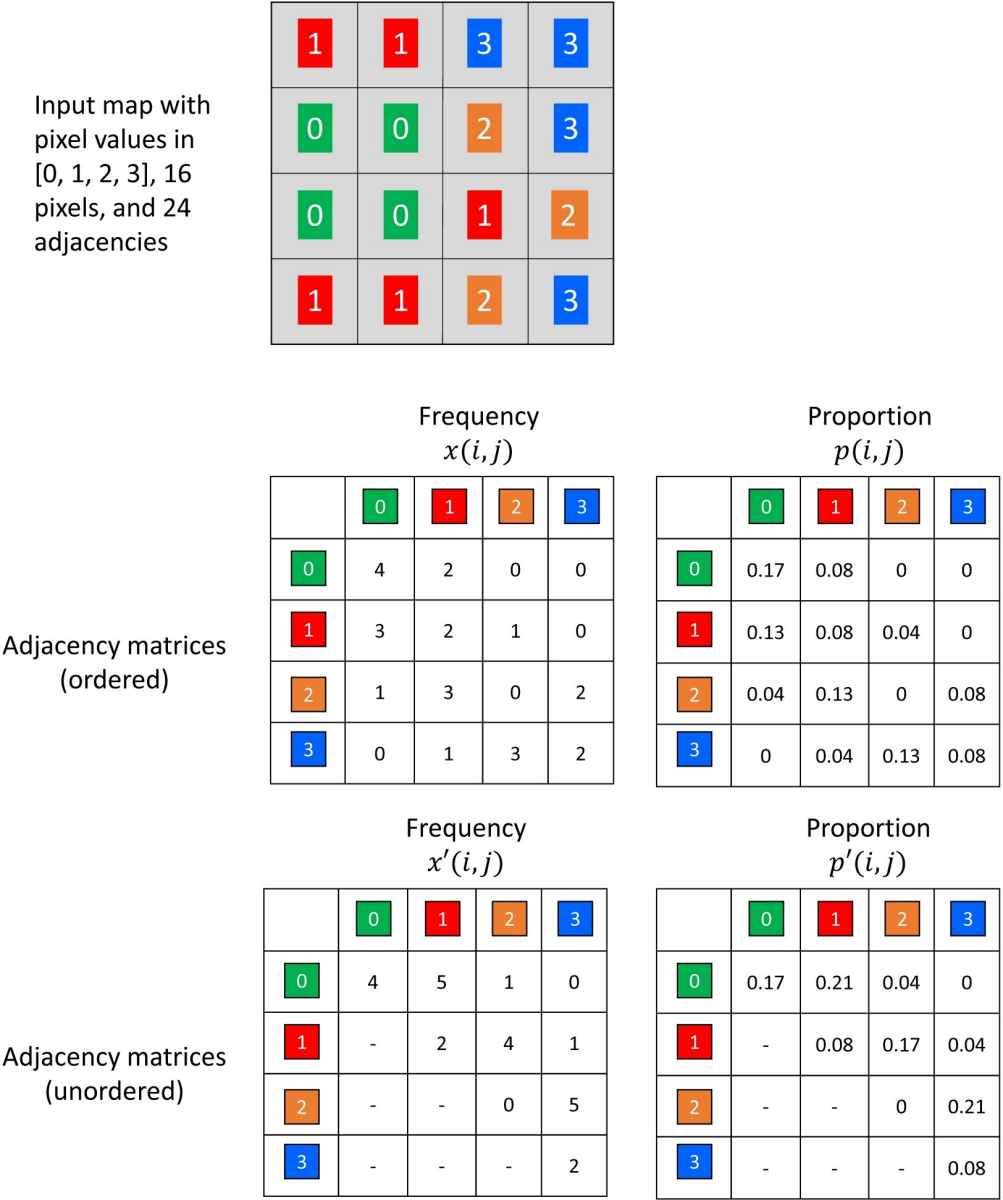
Illustration of ordered and unordered adjacency matrices for a 4 pixel x 4 pixel map containing nominal or ordinal data (top). Using a 2-neighbor rule defined as “one pixel below or one pixel to the right,” adjacencies are tabulated to form an ordered adjacency matrix (middle); an unordered adjacency matrix is formed by collapsing the ordered matrix across the main diagonal (bottom). The notation follows that in Table 1. Proportions are rounded to two decimal places.

To illustrate basic concepts, a 4 x 4 map contains 16 pixels and 24 adjacencies (Fig 1). Depending on the metric, the moving window algorithm tracks the changing pixel values in a window (summarized in a frequency distribution) and/or the changing pixel value adjacencies in a window (summarized in an adjacency matrix, also known as a co-occurrence matrix). Within an adjacency matrix, the rows and columns indicate the pixel values that are adjacent, and the elements of the matrix indicate the frequencies of each type of adjacency (Fig 2). In *GraySpatCon*, adjacency matrices are constructed by counting each adjacency in a window once to construct an ordered adjacency matrix; an unordered adjacency matrix is constructed by collapsing the ordered matrix across the main diagonal (Fig 2). Where appropriate, *GraySpatCon* provides two versions of metrics reflecting the difference between ordered and unordered adjacencies.

Using the notation in Table 1, the *GraySpatCon* metrics are defined in Table 2 where the metric numbers and names follow the nomenclature of the GuidosToolbox (GTB) and GuidosToolbox Workbench (GWB) applications (see below). For consistency with common practice in the image processing literature, the metric definitions assume that numeric data are quantized (“binned”) as integer values in the range [0, 100]; nominal input data can take on any values in that range. Because quantized numeric data are ordinal and therefore categorical, all the metrics can be calculated for a given input map. However, not all metrics are meaningful for all types of input data (Table 2); for example, the correlation metric is meaningless when using nominal data, and the landscape mosaic metric is meaningless when using numeric data. The development philosophy is to provide a generic tool that is potentially applicable to a wide range of investigations. We assume knowledgeable users will select appropriate metrics for a specific circumstance and interpret the results in that context. We recognize the algebraic and geometric correlations among many of the metrics, and we do not advocate mindless calculation or application of them.

**Table 1:** Notation for metric computation.

Notation	Definition	Notes
Notation for metrics based on the frequency of pixel values (gray levels)		
*i*	Gray level of the frequency array	$i\in \left\{ \begin{array}{c} (0,100)if\;zero\;gray\;level\;is\;included\\ (1,100)if\;zero\;gray\;level\;is\;excluded \end{array}\right.$
*N*_*g*_	Number of gray levels	
*x*(*i*)	Element *i* in the frequency array	
*p*(*i*)	$\frac{x(i)}{\sum_{\;i}\;x(i)}$	
*μ*	$\sum_{\;i}\left[i∙p(i)\right]$	
*σ* ^2^	$\sum_{\;i}\left[{\left(i-\mu \right)}^{2}∙p(i)\right]$	
*t*_1_	User-selected gray level	Target code 1
Notation for metrics based on the frequency of pixel value (gray level) adjacencies		
*i and j*	Gray levels *i* (row) and *j* (column) in the adjacency matrix	$i,j\in \left\{ \begin{array}{c} (0,100)if\;zero\;gray\;level\;is\;included\\ (1,100)if\;zero\;gray\;level\;is\;excluded \end{array}\right.$
*N*_*g*_	Number of gray levels in the adjacency matrix	
*R*	Range of gray levels	
*x*(*i*,*j*)	Element *i*, *j* in the adjacency matrix	Ordered adjacencies
*x*′(*i*,*j*)	$\left\{ \begin{array}{c} x(i,j)+x(j,i),if\;i\neq j\\ x(i,j),if\;i=j \end{array}\right.$	Unordered adjacencies
*p*(*i*,*j*)	$\frac{x(i,j)}{\sum_{\;i}\;\sum_{\;j}\;x(i,j)}$	
*p*′(*i*,*j*)	$\frac{x^{\prime }(i,j)}{\sum_{\;i}\;\sum_{\;j}\;x(i,j)}$	
*p*_*x*_(*i*)	$\sum_{j}\;p(i,j)$	
*p*_*y*_(*j*)	$\sum_{i}\;p(i,j)$	
*μ*_*x*_	$\sum_{\;i}\left[i∙p_{x}(i)\right]$	
*μ*_*y*_	$\sum_{\;j}\left[j∙p_{y}(j)\right]$	
$\sigma_{x}^{2}$	$\sum_{i}\left[{\left(i-\mu_{x}\right)}^{2}∙p_{x}(i)\right]$	
$\sigma_{y}^{2}$	$\sum_{j}\left[{\left(j-\mu_{y}\right)}^{2}∙p_{y}(j)\right]$	
*p*_*x*−*y*_(*k*)	$\sum_{\;i}\;\sum_{\;j\;where|i-j|=k}\;p(i,j)$	$k\in \left\{ \begin{array}{c} (0,100)\;if\;zero\;gray\;level\;is\;included\\ (0,99)\;if\;zero\;gray\;level\;is\;excluded \end{array}\right.$
*p*_*x*+*y*_(*k*)	$\sum_{\;i}\;\sum_{\;j\;where\;i+j=k}\;p(i,j)$	$k\in \left\{ \begin{array}{c} (0,200\;)\;if\;zero\;gray\;level\;is\;included\\ (2,200)\;if\;zero\;gray\;level\;is\;excluded \end{array}\right.$
*N*_*k*_	Number of *k* levels in the adjacency matrix	
*t*_1_	User-selected gray level	Target code 1
*t*_2_	User-selected gray level	Target code 2
k*	User-selected *k* level	For the kContagion metric only.

**Table 2:** *GraySpatCon* metrics.

Metric (1)	Short name (2)	Description (3)	IT (4)	OT (5)
1	Mean	Mean: ∑_*i*_ [*i*∙*p*(*i*)]	O	R [0,100]
2	EvennessOrderedAdj	Evenness (ordered adjacencies) [ref. 41]: $\frac{\left[M4\right]}{2∙\log N_{g}},$ *for N*_*g*_>1	A	R [0,1]
3	EvennessUnorderedAdj	Evenness (unordered adjacencies) [ref. 42]: $\frac{\left[M5\right]}{\left[\log(N_{g}^{2}+N_{g})-\log2\right]},$ *for N*_*g*_>1	A	R [0,1]
4	EntropyOrderedAdj	Entropy (ordered adjacencies) [ref. 43]: $-\sum_{\;i}\;\sum_{\;j\;}\left[p(i,j\right)∙\log p(i,j)],\;for\;p(i,j)>0$	A	R
5	EntropyUnorderedAdj	Entropy (unordered adjacencies) [ref. 42]: $-\sum_{\;i}\;\sum_{\;j\geq i\;}\left[p^{\prime }(i,j\right)∙\log\;p^{\prime }(i,j)],\;for\;p(i,j)>0$	A	R
6	DiagonalContagion	Diagonal Contagion [ref. 44]: ∑_*i*_ *p*(*i*,*i*)	A	R [0,1]
7	ShannonDiversity	Shannon Diversity (gray levels): $-\sum_{\;i\;}\left[p(i\right)∙\log\;p(i)],\;for\;p(i)>0$	A	R
8	ShannonEvenness	Shannon Evenness (gray levels): $\frac{\left[M7\right]}{\log Ng},$ *for N*_*g*_>1	A	R [0,1]
9	Median	Median: *median* [*x*(*i*)]	O	I [0,100]
10	GSDiversity	Gini-Simpson Diversity (gray levels): 1−∑_*i*_*p*(*i*)^2^	A	R [0,1]
11	GSEvenness	Gini-Simpson Evenness (gray levels): $\frac{\left[M10\right]}{1-(1/N_{g})},$ *for N*_*g*_>1	A	R [0,1]
12	EquitabilityOrderedAdj	Equitability (ordered adjacencies) [ref. 45]: $\frac{\left[M14\right)}{1-(1/N_{g}^{2})},$ *for N*_*g*_>1	A	R [0,1]
13	EquitabilityUnorderedAdj	Equitability (unordered adjacencies) [ref. 46]: $\frac{\left[M15\right)}{1-[\;2/\left(N_{g}^{2}+N_{g}\right)]},$ *for N*_*g*_>1	A	R [0,1]
14	DiversityOrderedAdj	Gini-Simpson Diversity (ordered adjacencies) [ref. 43, 45]: $1-\sum_{\;i}\;\sum_{\;j\;}\left[p(i,j\right)∙p(i,j)]$	A	R [0,1]
15	DiversityUnorderedAdj	Gini-Simpson Diversity (unordered adjacencies): $1-\sum_{\;i}\;\sum_{\;j\geq i\;}\left[p^{\prime }(i,j\right)∙p^{\prime }(i,j)]$	A	R [0,1]
16	Majority	Majority: *i*|*p*(*i*) = *maximum*[*p*(*i*)]	A	I [0,100]
17	LandscapeMosaic19	Landscape Mosaic (19 classes; see LM product sheet): $Ternary\;classification\;of\;[\;p(1),p(2),p(3)],\;if\;f\;\sum_{i=1}^{3}\;p(i)=1$	N	N [1,19]
18	LandscapeMosaic103	Landscape Mosaic (103 classes; see LM product sheet): $Ternary\;classification\;of\;[\;p(1),p(2),p(3)],\;if\;f\;\sum_{i=1}^{3}\;p(i)=1$	N	N [1,103]
19	NumberGrayLevels	Number of Gray Levels: *Ng*	A	I [1,100]
20	MaxAreaDensity	Maximum Area Density: *maximum*[*p*(*i*)]	A	R [0,1]
21	FocalAreaDensity	Focal Area Density: *p*(*t*_1_)	A	R [0,1]
22	FocalAdjT1	Focal adjacency (t_1_): $\frac{\sum_{\;j}x^{\prime }(t_{1},j)}{\sum_{\;i}\;\sum_{\;j\;}\;x(i,j)}$	A	R [0,1]
23	FocalAdjT1andT2	Focal adjacency (t_1_ and t_2_) [ref. 47]: $\left\{ \begin{array}{c} \frac{x(t_{1},t_{2})+x(t_{2},t_{1})}{\sum_{i}\;\sum_{j}x(i,j)},\;if\;t_{1}\neq t_{2}\;\\ \frac{x(t_{1},t_{1})}{\sum_{i}\;\sum_{j}x(i,j)},if\;t_{1}=t_{2} \end{array}\right.$	A	R [0,1]
24	FocalAdjT1givenT2	Focal adjacency (t_2_ given t_1_) [ref. 48, 49]: $\left\{ \begin{array}{c} \frac{x(t_{1},t_{2})+x(t_{2},t_{1})}{\sum_{\;j}x^{\prime }(t_{1},j)},\;if\;t_{1}\neq t_{2}\;\\ \frac{x(t_{1},t_{1})}{\sum_{\;j}x^{\prime }(t_{1},j)},\;if\;t_{1}=t_{2}\; \end{array}\right.$	A	R [0,1]
25	StandardDeviation	Standard Deviation (population estimator): $\sqrt{\sum_{\;i}\left[{\left(i-\mu \right)}^{2}∙p(i)\right]}$	O	R [0,100]
26	CoefficientVariation	Coefficient of Variation: $100∙\left(\frac{\left[M25\right]}{\mu }\right),$ *for μ*>0	O	R
27	Range	Range: *maximum*(*i*)−*minimum*(*i*)	O	I [0,100]
28	Dissimilarity	Dissimilarity] [ref. 50]: $\sum_{\;i}\;\sum_{\;j\;}\left[|i-j|∙p(i,j\right)\;$	O	R
29	Contrast	Contrast [ref. 43, 51]: $\sum_{\;i}\;\sum_{\;j\;}\left[{\left(i-j\right)}^{2}∙p(i,j\right)]$	O	R
30	UniformityOrderedAdj	Uniformity (ordered adjacencies) [ref. 43]: $\sum_{\;i}\;\sum_{\;j\;}\left[p(i,j\right)∙p(i,j)]$	A	R [0,1]
31	UniformityUnorderedAdj	Uniformity (unordered adjacencies): $\sum_{\;i}\;\sum_{\;j\geq i\;}\left[p^{\prime }(i,j\right)∙p^{\prime }(i,j)]$	A	R [0,1]
32	Homogeneity	Homogeneity [ref. 43, 50]: $\sum_{\;i}\;\sum_{\;j\;}\left[\frac{p(i,j)}{1+{\left(i-j\right)}^{2}}\right]$	O	R [0,1]
33	InverseDifference	Inverse Difference [ref. 51]: $\sum_{\;i}\;\sum_{\;j\;}\left[\frac{p(i,j)}{1+|i-j|}\right]$	O	R [0,1]
34	SimilarityRMax	Similarity (R = 100) [ref. 52]: $1-\sum_{\;i}\;\sum_{\;j\;}\left[\frac{\left|i-j)∙p(i,j\right)}{100}\right]$	O	R [0,1]
35	SimilarityRGlobal	Similarity (R = global range) [ref. 52]: $1-\sum_{\;i}\;\sum_{\;j\;}\left[\frac{\left|i-j)∙p(i,j\right)}{global\;range}\right]$	O	R [0,1]
36	SimilarityRWindow	Similarity (R = window range) [ref. 52]: $1-\sum_{\;i}\;\sum_{\;j\;}\left[\frac{\left|i-j)∙p(i,j\right)}{window\;range}\right]$	O	R [0,1]
37	DominanceOrderedAdj	Dominance (ordered adjacencies) [ref. 50]: *maximum*[*p*(*i*,*j*)]	A	R [0,1]
38	DominanceUnorderedAdj	Dominance (unordered adjacencies): *maximum*[*p*′(*i*,*j*)]	A	R [0,1]
39	DifferenceEntropy	Difference Entropy [ref. 43]: $-\sum_{\;k}{[p}_{x-y}(k)∙\log\;p_{x-y}(k)],\;for{\;p}_{x-y}(k)>0$	O	R
40	DifferenceEvenness	Difference Evenness [ref. 46]: $\frac{\left[M39\right]}{\log N_{k}},$ *for N*_*k*_>1	O	R [0,1]
41	SumEntropy	Sum Entropy [ref. 43]: $-\sum_{\;k}{[p}_{x+y}(k)∙\log\;p_{x+y}(k)],\;for{\;p}_{x+y}(k)>0$	O	
42	SumEvenness	Sum Evenness [ref. 46]: $\frac{\left[M41\right]}{\log N_{k}},$ *for N*_*k*_>1	O	R [0,1]
43	AutoCorrelation	Autocorrelation [ref. 50]: $\sum_{\;i}\;\sum_{\;j\;}\left[(i∙j)∙p(i,j\right)]$	O	R
44	Correlation	Correlation [ref. 43, 51]: $\sum_{\;i}\;\sum_{\;j\;}\left[\left(\frac{i-\mu_{x}}{\sigma_{x}})∙(\frac{j-\mu_{y}}{\sigma_{y}})∙p(i,j\right)\right],\;for\;\sigma_{x},\sigma_{y}>0$	O	R [–1,1]
45	ClusterShade	Cluster Shade [ref. 53]: $\sum_{\;i}\;\sum_{\;j\;}\left[\;{\left(i+j-\mu_{x}-\mu_{y}\right)}^{3}∙p(i,j)\right]$	O	R
46	ClusterProminence	Cluster Prominence [ref. 53]: $\sum_{\;i}\;\sum_{\;j\;}\left[\;{\left(i+j-\mu_{x}-\mu_{y}\right)}^{4}∙p(i,j)\right]$	O	R
47	RootMeanSquare	Root Mean Square: $\sqrt{\sum_{\;i}\left[i^{2}∙p(i)\right]}$	O	R
48	AverageAbsDeviation	Average Absolute Deviation: $\sum_{\;i}\left[|i-\mu |∙p(i)\right]$	O	R
49	kContagion	*k*-contagion [ref. 46]: $\sum_{k=0\;}^{k^{*}}p_{x-y}(k)$	O	R [0,1]
50	Skewness	Skewness: $\frac{\sum_{\;i}\left[{\left(i-\mu \right)}^{3}∙p(i)\right]}{\sigma^{3}},$ *for σ*>0	O	R
51	Kurtosis	Kurtosis: $\frac{\sum_{\;i}\left[{\left(i-\mu \right)}^{4}∙p(i)\right]}{\sigma^{4}},$ *for σ*>0	X	R

Columns 1–2: the metric number and short name as used in the parameter popup window (GTB) and the text parameter file (GWB). Column 3: metric description (“[MX]” in equation indicates substitution of metric number X; “[ref. X]” refers to citation number X). Column 4: input data type (“A” = all, “N” = nominal, “O” = ordinal). Column 5: output data type and bounding range (if any] (“I” = integer, “N” = nominal, “R” = real).

Some of the metric definitions in Table 2 differ slightly from other published definitions. This is due partly to differences in the handling of input pixels which are coded as zero (*GraySpatCon* optionally omits those pixels from calculations), and in the method used to construct adjacency matrices (e.g., ordered, unordered, or symmetric). In addition, certain metrics have the same name in the landscape ecology and image processing literature when different formulas are used, or different names when the formulas are the same.

The default output precision in *GraySpatCon* is 32-bit floating point, with optional conversion to 8-bit integer precision. *GraySpatCon* optionally performs a global analysis using the entire extent of the input data area, in which case the output is a text file instead of a map. The moving window algorithm in *GraySpatCon* is parallelized using OpenMP and by default, will use all cores available in the operating system. The *GraySpatCon* memory requirement is approximately five times the number of pixels (bytes) in the input map (e.g., ~20 MB RAM for a map of size 2000 x 2000 pixels).

**Fig 3:**
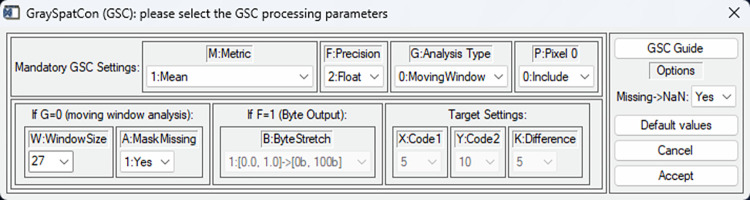
Popup window used to set *GraySpatCon* parameters in the GuidosToolbox application (S1 File).

While it is possible for a user to compile and execute a stand-alone application (S1 File), we strongly suggest to first use *GraySpatCon* via its implementation in the free and open-source image analysis software application GTB (GuidosToolbox) [54]. GTB contains a wide variety of generic raster image processing routines, which are packaged into an interactive desktop application for either Linux, macOS, or MS-Windows. In GTB, the authors have implemented *GraySpatCon* with a dedicated popup window (Fig 3). This GUI-interface is designed to provide the most intuitive and user-friendly link to *GraySpatCon*, facilitating the correct interaction and selection of the *GraySpatCon* parameter settings. Batch-mode, or automatic processing of a series of images, is also available in GTB. The implementation of *GraySpatCon* in GTB is described further in the *GraySpatCon* Guide (S1 File), which also contains important usage information and general instructions for a *GraySpatCon* stand-alone application. For workflow applications in a Linux desktop or server environment, *GraySpatCon* is also implemented as the GWB_GSC module in the free and open-source software application GWB (GuidosToolbox Workbench) [55]. If computer memory is limited, large images can be processed efficiently by using the GWB_SPLITLUMP module.

## Results and discussion

### Examples

**Fig 4:**
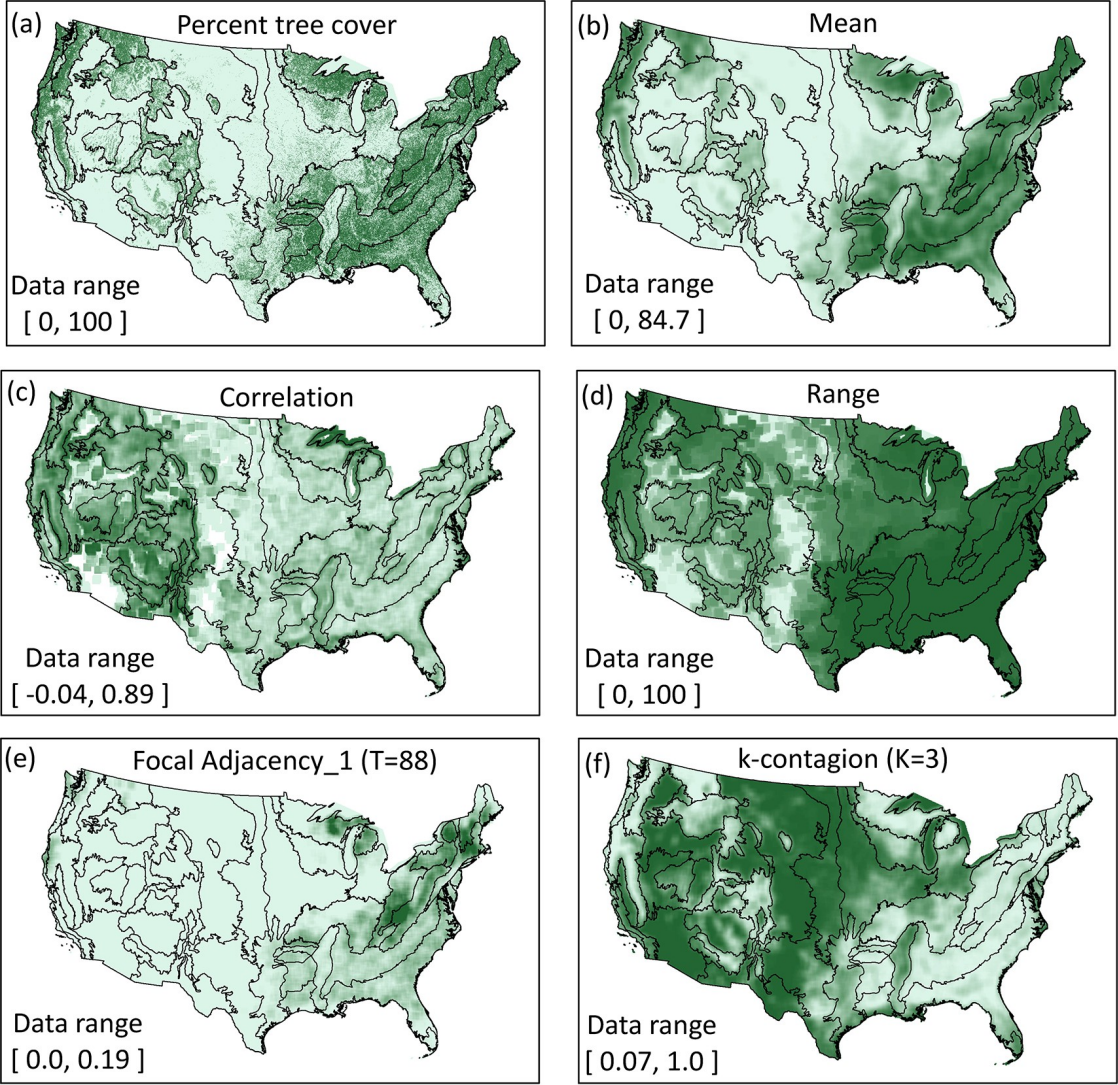
Illustration of *GraySpatCon* map output for five metrics applied to a map of percent tree cover for the conterminous United States. Five landscape pattern metrics were mapped using a window size of 31x31 pixels (5675 km^2^). The panels show the input data (a) [56] and output data (b–f) for five metrics (respectively, metric numbers 1, 44, 27, 22, 49 in Table 2). Darker colors indicate larger values, ecoregion boundaries [57] are shown for comparisons, and the data range in each panel is indicated.

We illustrate the functionality of *GraySpatCon* with five landscape pattern metrics applied to a map of percent tree cover in the conterminous United States [56] resampled to a resolution of 2430m (Fig 4 and S2 File). The five metrics include first-order (mean, range) and second-order (correlation, k-contagion) metrics, and a second-order metric targeted at adjacencies involving a specific input pixel value (focal adjacency_1). While a visual comparison of the output maps may suggest the five metrics capture different aspects of the pattern of percent tree cover, the choice of metrics in a specific study naturally depends on the circumstances and the results and interpretations will vary accordingly. Ultimately the perception of pattern depends on the observer. For example, investigations of habitat patterns for different species should use appropriate habitat data and metrics which describe the aspects of pattern that are believed to be important from the perspective of each species [4]. Since all potential observers cannot be known in advance, *GraySpatCon* is designed to quantify and map many pattern metrics in a consistent way such that comparisons among different studies may be facilitated.

**Fig 5:**
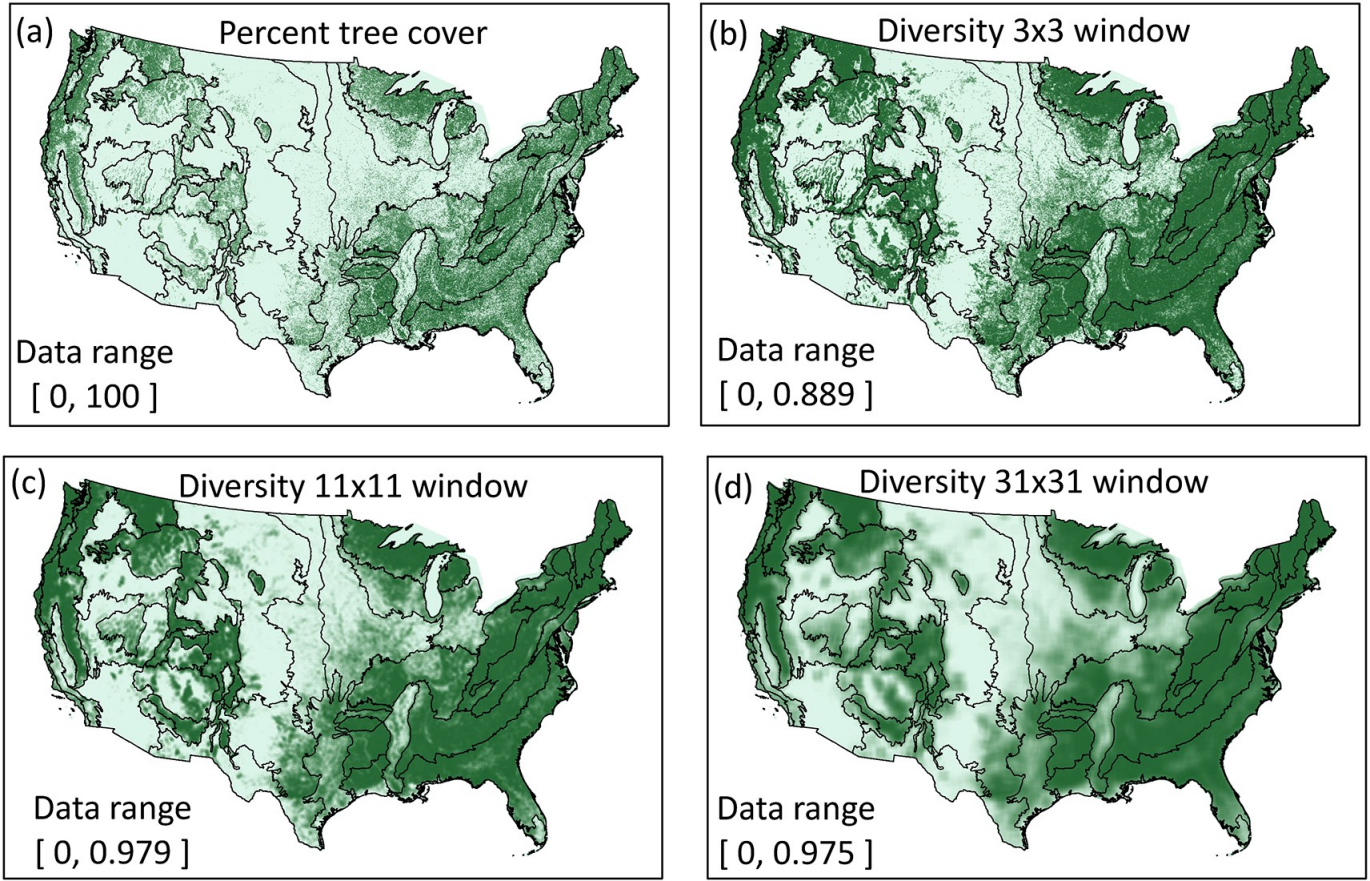
Illustration of changing the observation scale (window size) for the Gini-Simpson diversity metric (Table 2; metric 10) as applied to a map of percent tree cover (a) [56]. The diversity of percent tree cover was mapped for the three indicated window sizes (b, c, d) corresponding to window areas of 53 km^2^, 714 km^2^, and 5675 km^2^). Darker colors indicate larger values, ecoregion boundaries [57] are shown for comparisons, and the data range in each panel is indicated.

**Fig 6:**
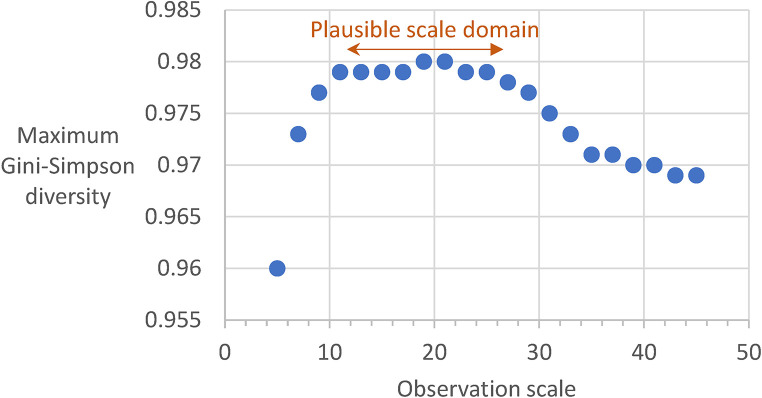
Illustration of a scalogram showing the maximum Gini-Simpson diversity metric over all windows, over a range of observation scales (window sizes) for a map of percent tree cover (c.f., Fig 5). The observation scale is indicated by the side length of the moving window, for example an observation scale of 15 corresponds to a window size of 15x15 pixels. The plausible scale domain is a range of observation scales over which the maximum value is relatively stable.

It is also well known that the perceived pattern depends on the observation scale. In ecology this is often translated to a requirement of selecting an observation scale which is appropriate for a specific ecological process or species [4]. In a broader view, ecologists are also interested in how pattern *per se* changes with observation scale in different landscapes; for example, “scalograms” [58] may suggest “scale domains” [59] over which pattern-process relationships may be stable. The “scale parameter” In *GraySpatCon* is only one aspect of observation scale–the window size which defines the spatial extent of the analysis. Fig 5 illustrates the effect of changing the window size on the Gini-Simpson diversity metric as applied to the map of percent tree cover. An example of a scalogram was constructed using 21 window sizes from 5x5 pixels to 45x45 pixels and plotting the maximum metric value (over all windows) versus window size (Fig 6). The stability of the maximum metric value in window sizes from 11x11 to 25x25 suggests a plausible scale domain, which could be tested for significance and subsequently compared to an ecological process which was measured at the same 21 observation scales.

The example in Fig 6 used the maximum metric value over all windows to illustrate a simple scalogram. While beyond the scope of this illustration, the multi-scale results for each location (i.e., each window) could be collectively analyzed to identify and map the locations of prevalent scalograms [60] and identify the scales and locations of transitions from global to local scaling relationships [61]. While the traditional patch-corridor-matrix metrics may be better suited for describing discrete patches, similar multi-scale procedures could be applied to nominal data to describe imputed patches in terms perimeter characteristics, juxtaposition, size, and spatial distribution [62, 63].

### Computational efficiency

Computational efficiency is an important aspect of landscape pattern analysis, especially when addressing problems requiring calculations for large or numerous input maps, or for many metrics or window sizes. A full comparison of all *GraySpatCon* metrics with other software is not possible because no other software implements all the *GraySpatCon* metrics. We selected a set of comparable metrics for illustration and conducted all trials in the R desktop environment [64] using the map of percent tree cover described above (S2 File). We compared *GraySpatCon* moving window analyses of four second-order Haralick [43] texture metrics with the R packages *glcm* [34] and *fastGLCM* [35] (Table 3). We also compared *GraySpatCon* global and moving window analyses of one second-order and two first-order metrics with the R package *landscapemetrics* [20] (Table 4). In the moving window comparisons *GraySpatCon* was substantially faster than all three alternatives. In the global analysis comparisons, *GraySpatCon* was substantially faster than *landscapemetrics* for two of the three metrics tested. While alternate software may be required for metrics not implemented in *GraySpatCon*, we believe these limited comparisons are a compelling argument to consider using *GraySpatCon* when there is a choice.

**Table 3:** Comparison of moving window execution times for four image texture metrics in *glcm*, *fastGLCM*, and *GraySpatCon*. The gray-scale input map (Fig 4A) is 1990 X 1289 pixels, the window size is 31x31 pixels, and the reported time is the mean of three trials.

	Software		
	*glcm* ^1^	*fastGLCM*	*GraySpatCon*
	Metric: homogeneity		
1 core	3.7 minutes	1.6 minutes	17.2 seconds
8 cores	--	27.4 seconds	4.1 seconds
	Metric: entropy		
1 core	4.9 minutes	1.7 minutes	48.3 seconds
8 cores	--	27.8 seconds	10.6 seconds
	Metric: contrast		
1 core	3.1 minutes	1.6 minutes	17.5 seconds
8 cores	--	26.9 seconds	4.2 seconds
	Metric: angular second moment		
1 core	2.9 minutes	1.6 minutes	16.9 seconds
8 cores	--	26.4 seconds	4.1 seconds

^1^*glcm* is single-core only.

Hardware: CPU– 2 x Xeon E5-1620 @ 3.7GHz; RAM– 64GB @ 1866MHz.

Platform: Windows10(x64); R version 4.1.3 (2022-03-10); RStudio 2022.02.1+461.

Software versions: *glcm* 1.6.5; *fastGLCM* 1.0.2; *GraySpatCon* 1.1.1.

Execution time was measured by the R function *Sys*.*time()* before and after the relevant function or program.

**Table 4:** Comparison of moving window and global analyses for *landscapemetrics* and *GraySpatCon*. The gray-scale input image (Fig 4A) is 1990 X 1289 pixels, the window size is 3x3 pixels, and the reported time is the mean of three trials. Multi-core times were not compared because *landscapemetrics* supports only single-core usage, and for practical reasons the smallest possible window size was used.

	Moving window (3 x 3)		Global analysis	
	Software		Software	
	*Landscapemetrics*	*GraySpatCon*	*Landscapemetrics*	*GraySpatCon*
Metric	lsm_l_pr	NumberGrayLevels	lsm_l_pr	NumberGrayLevels
	1.2 hours	0.4 seconds	0.18 seconds	0.17 seconds
Metric	lsm_l_contag	EvennessOrderedAdj	lsm_l_contag	EvennessOrderedAdj
	1.5 hours	19.3 seconds	0.36 seconds	0.17 seconds
Metric	lsm_l_siei	GSEvenness	lsm_l_siei	GSEvenness
	6.9 hours^1^	0.5 seconds	8.11 seconds	0.16 seconds

^1^One trial completed in this time; two others were stopped after 3 hours.

Hardware: CPU– 2 x Xeon E5-1620 @ 3.7GHz; RAM– 64GB @ 1866MHz.

Platform: Windows10(x64); R version 4.1.3 (2022-03-10); Rstudio 2022.02.1+461.

Software versions: *landscapemetrics* 1.5.6; *GraySpatCon* 1.1.1.

Execution time was measured by the R function *Sys*.*time()* before and after the relevant function or program.

## Conclusion

Landscape ecologists use a variety of software and metrics to quantify and map landscape patterns using raster data, but there are relatively few open-source software alternatives for conducting many of the analyses commonly used to explore pattern-process relationships, particularly software that supports analysis of numeric input data. We developed *GraySpatCon* to quantify and map a wide range of landscape pattern metrics using categorical or numeric input data, and our comparisons with alternate software indicated a significant advantage in computational efficiency. We implemented *GraySpatCon* in the popular GuidosToolbox desktop application so that novice users can interactively explore its capabilities, and in the GuidosToolbox Workbench so that experienced users can easily integrate its capabilities in computationally intensive workflows. Alternatively, binary executable versions of *GraySpatCon* are available for three popular operating systems for implementation in other desktop applications such as R (S2 File). For developers, the source code for *GraySpatCon* is distributed on GitHub under CC0 (CC0 1.0 Universal [CC0 1.0] Public Domain Dedication) (S2 File). Through these efforts we hope to stimulate increased attention to application of numeric maps in ecology and related fields, and consistent treatment of nominal and numeric data where appropriate.

## Supporting information

S1 File The Guide describes implementation in the GuidosToolbox application and provides important usage notes. (PDF)

S2 File This archive contains example input maps and R scripts used for examples and comparisons, and instructions to download *GraySpatCon* source code and binary executable files for 64-bit Linux, macOS, and MS-Windows operating systems. (ZIP)
