# Supplementary material for: Mapping landscape ecological patterns using numeric and categorical maps
Source: PLoS One. 2023 Nov 15;18(11):e0291697. doi: 10.1371/journal.pone.0291697 (PMC10651036; doi:10.1371/journal.pone.0291697)
Supplement: S1 File — (PDF) [file pone.0291697.s001.pdf]

GraySpatCon Guide

Kurt Riitters, Peter Vogt, April 2023.

This document provides information on features, options, input data requirements, parameter settings and the use of the open-source spatial convolution program ***GraySpatCon*** (Gray-level Spatial Convolution, version 1.1.1). In addition to the standalone version, this document also describes the *GraySpatCon* implementation in various software packages.
Important note: This is a living document. The latest version of this document, and the most recent version of the *GraySpatCon* source code, is available on GitHub.

Contents.

1. General description
2. Important usage notes
3. *GraySpatCon* parameters
4. *GraySpatCon* metrics
5. Compiling and using *GraySpatCon* in standalone mode
6. Literature cited

1. General description

GraySpatCon implements a moving window analysis of a two-dimensional image (or a map) comprised of pixels (grid cells). It moves a square window across the image, one pixel at a time, accumulating and discarding information along the way. Based on the pixel values in the window on the input image at a given pixel location, a metric is calculated and assigned to that location on the output image. Thus, the output pixel value codes the input context of that pixel location, and the spatial resolution of the input image is preserved. The spatial scale of the analysis is defined by the size of the window[1]. All *GraySpatCon* metrics are calculated from (a) the frequencies of different input pixel values in the window, and/or (b) the pixel value adjacency matrix for the window, which contains the frequencies of different types of pixel value adjacencies in the window. Each adjacency in either a horizontal or vertical direction is counted once[2], and adjacencies at the boundary of the window are ignored. For example, a 3x3 window contains 9 pixels and 12 adjacencies (Fig. 1).

Figure 1. Example 3x3 pixel window containing 9 pixels (numbered in black font) and 12 adjacencies (red font).

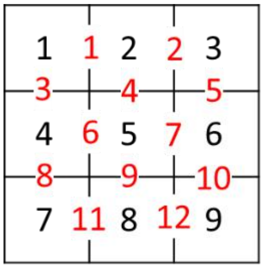

[1] Other aspects of scale are defined by the input dataset – spatial resolution (pixel size), spatial extent (image size), and thematic resolution (image legend).
[2] *GraySpatCon* uses the 2-neighbor rule, does not double-count adjacencies (e.g., by the 4-neighbor rule), and does not recognize adjacencies in diagonal directions. The 2-neighbor rule is equivalent to the argument “shift = list(c(0,-1), c(1,0))” in the R *glcm* package (https://www.rdocumentation.org/packages/glcm/versions/1.6.5).

2. Important usage notes

While *GraySpatCon* can be compiled and used in standalone mode (see Section 5) or within a packaged software (see Section 3), we strongly suggest to first use *GraySpatCon* via its implementation in the free image analysis software GTB (GuidosToolbox, [ref. 12]). GTB contains a wide variety of generic raster image processing routines, which are packaged into an interactive desktop application for either Linux, macOS, or MS-Windows. The instructions in this Guide refer specifically to usage with GTB.

2.1. Missing values

2.1.1 Moving window analysis

In *GraySpatCon*, the input image is buffered on all four sides with missing pixel values, where the buffer size is selected so that a full window can be centered on every pixel in the input image[3]. User-defined missing pixels can also occur within the image area. The metrics are calculated from the non-missing pixels in a window. The result is stored as the location of the output pixel in the center of the window, even if the user has defined that input pixel as missing. For example, suppose that ocean water is defined as missing on an input land cover image. The pattern metric image may have metric values where the ocean water is near the coastline because a portion of the window is not ocean water. *GraySpatCon* will optionally convert all pixels that are missing input data to missing output data.

GraySpatCon always produces a missing value if there is insufficient information in the window to perform a given calculation. For metrics calculated from pixel value frequencies, this can happen when all pixels in the window are missing. For metrics calculated from the frequencies of pixel value adjacencies, this can happen when all requested adjacencies within the window are missing; an adjacency is missing if either of the two adjacent pixels is missing. Thus, it is possible for a window which contains a given pixel value to have no adjacencies for that pixel value, for example a checkerboard image with only one pixel value separated by missing pixels. Adjacencies at the boundary of a window are ignored. Some metrics may yield missing values if an arithmetic function cannot be performed, or if there is an insufficient number of unique pixel values, or number of unique pixel adjacencies (see Section 4).

2.1.2 Global analysis

The metrics are calculated from the non-missing pixels in the entire data area. Like a moving window analysis, metric values may be missing if the metric cannot be calculated from the global data area.

2.1.3 Missing values on input

The input image pixel value 255 is treated as missing data. Data values are integers in [0, 100]. Optionally, the pixel value 0 will also be treated as missing data. All other pixel values will cause an error and exit.

---

[3] If w is the side length of the window, the buffer width is $(w-1)/2$ pixels. For a pixel at the corner of a rectangular image, at least ¾ of the window is in the buffer area. The buffer area is removed before writing the output image.

2.1.4 Missing values on output

For 32-bit (float) output, the missing value is -0.01, except for metrics 44, 45, and 50 that may take on a negative value (see Section 4), in which case the missing value is -9000000.0. For 8-bit (byte) output the missing value is always 255.

Image viewers or GIS applications apply different schemes to visualize digital maps. They either show the actual data range from the minimum to the maximum value, or automatically apply a contrast enhancement (auto-stretching) for the data range. Moreover, not all applications account for the NODATA metadata information that may be specified in the GeoTIFF header. Depending on the image viewer application, the color scheme used to render the map may not provide a sufficient color range to differentiate between missing and non-missing values, which may result in all the non-missing pixels to appear in the same color. In GTB, this issue can be addressed via an additional post-processing step, using the switch *Missing -> NaN*, see right panel in Figure 2, having the following options:

- "Yes": will assign all NODATA pixels the value of NaN. This step will resolve output display issues and show consistent results across all image viewers. When saving a *GraySpatCon* image, GTB will write out the original *GraySpatCon* values for those pixels that were manually assigned to NaN via the GTB popup window.
- "No": will use the unmodified *GraySpatCon* output image. When saving a *GraySpatCon* image, GTB will add the respective NODATA information in the GeoTIFF header.

With either of these options, any user can then make necessary adjustments to amend the missing data value to either the original *GraySpatCon* value or any other value that is suitable for the image viewer software application.

While all input images are rectangular, the non-missing data area may be irregular shape (e.g., a map) within a bounding rectangle. In that case, metrics will be mapped for areas of missing input data where the window extends into the non-missing data area. The result is an artifactual enlarging of the apparent non-missing data area on the output map. This artifact is optionally removed by "masking" the output to retain only the pixels which are non-missing on the input map.

2.2. Hardware requirements

Available RAM (GB)[4]: Approximately 5 x image size in GB = 5 x number of rows x number of columns / 1024^3. For example, the RAM requirements for processing an image having 100,000 rows and 75,000 columns: uncompressed image size in GB = 100000 x 75000 / 1024^3 = 6.98492 GB. With a factor of 5, the *GraySpatCon* processing requires approximately 35 GB of available RAM. Please note that your access to the system available free RAM may be limited on a multi-user system.

On multi-core machines, all cores that are available within the executing shell will be used (default). To use fewer cores, perform the optional environment setting (see Section 5) before executing *GraySpatCon*.

---

[4] It is strongly recommended to NOT use disk swap space for large images because I/O speed is a major constraint. For large images, it is recommended to use the module GWB_SPLITLUMP to run GWB_GSC on a set of overlapping strips of input data.

2.3. Input data

Input image data must be 8-bit unsigned integers in the range [0, 100], or 255 to indicate missing data. The input data can be one of three types:

- Dichotomous. Also known as binary, having two mutually exclusive categories. For example, ownership: "yes" or "no."
- Nominal. More than two categories without order or sequence. For example, place of living in the US by state (50 categories)
- Ordinal. At least two categories that are ordered in equal intervals. For example, percent vegetation (continuous in [0.0, 100.0]) which is quantized ("binned") into discrete categories (ordinal in [0, 100]).

To process dichotomous or nominal input data, the pixel values can be any integer in [0, 100]. To process continuous (real number) data, the input image must be rescaled and quantized ("binned") into ordinal integer classes in the range [N1, N2] where $N1 \geq 0$ and $N2 \leq 100$.

2.4. Metric selection for different input data types

The metrics in *GraySpatCon* can be calculated for dichotomous, nominal, and/or ordinal input data because all three data types are represented as integers. However, individual metrics are meaningless when applied to an incompatible data type. For example, the correlation metric is meaningless for nominal input data. The user must ensure that selected metrics are compatible with the input data type (see Section 4).

2.5 Output files

For a global analysis, the result is a plain text file with the parameters used and the global metric value. For a moving window (non-global) analysis, the result is a set of two files: a) a plain text file with the parameters used, and b) a (Geo)TIFF formatted image of the same dimension as the input image but showing the selected metric value at pixel level and in the format specified by the *GraySpatCon* processing parameters, for example, see here.

Metrics are calculated as integer, nominal, or floating-point values. For the metrics which are floating-point values, *GraySpatCon* rounds the calculated value to 5 decimal digits (1E-05) for output of either float values or rescaled byte values.

3. GraySpatCon parameters

GraySpatCon requires a set of parameters, which are specified in the file gsc-parameters.txt. Each line has a parameter code (a letter), a space, and a parameter value *X* (a number). Parameters that are not needed for the selected metric are ignored.

While *GraySpatCon* can be used in standalone mode (see Section 5), or within a packaged software (see below), we strongly suggest to first use *GraySpatCon* via its implementation in the free image analysis software GTB (GuidosToolbox, [ref. 12]). GTB contains a wide variety of generic raster image processing routines, which are packaged into an interactive desktop application for either Linux, macOS, or MS-Windows. In GTB, the authors have implemented *GraySpatCon* with a dedicated popup window (Fig. 2). This GUI-interface is designed to provide the most intuitive and user-friendly link to *GraySpatCon*, facilitating the correct interaction and selection of the *GraySpatCon* parameter settings.

Figure 2. Popup window used to set *GraySpatCon* parameters in GTB.

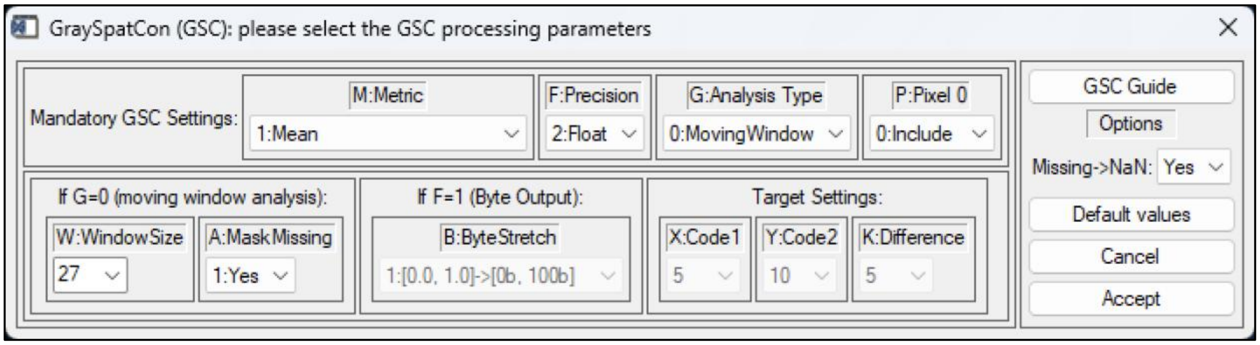

In GTB, the file gsc-parameters.txt is written automatically after collecting all *GraySpatCon* parameter settings via the dedicated popup window. These settings are then applied to the currently loaded image (GTB menu: Image Analysis → Pattern → GraySpatCon), and the result can be queried directly in the viewport of GTB. Moreover, a series of images can be processed with *GraySpatCon* via the batch-processing utility (GTB menu: File → Batch Process → Pattern → GraySpatCon) using the same GUI-interface (Fig. 2).

Alternative to GTB, *GraySpatCon* is also implemented in the following software applications:

- GWB: the GuidosToolbox Workbench (GWB, [ref. 13]) contains the most popular image analysis modules from GTB, setup as command line-only scripts for automated mass-processing on Linux 64bit servers. Here, the GWB module GWB_GSC applies the *GraySpatCon* parameter settings specified in the file gsc-parameters.txt to all (Geo)TIFF-images placed in the directory *input/*.
- R: the *GraySpatCon* plugin for R/RStudio is in development …
- QGIS: the *GraySpatCon* plugin for Quantum GIS is in development…
- ArcGIS: the *GraySpatCon* plugin for ArcGIS is in development…
- …

3.1 *GraySpatCon* parameter listing:

Required:

- M x = *GraySpatCon* metric: 1, 2, 3, …, 51 (see Section 4)
- F x = output map precision: 1 = 8-bit, 2 = 32-bit float. Note that F 2 *must* be used for metrics 44, 45, 50.
- G x = analysis type: 0 = moving window analysis; 1 = global (entire map extent) analysis

Optional:

- P x = exclude input pixels with value zero: 0 = no, 1 = yes

Required if G 0:

- W x = window size: the number of pixels on the side of a x*x window (e.g., W 5 for a 5x5 window), must be an odd and positive integer number > 1 (e.g., 3,5,7,9...) , maximum < x or y dimension of input map

Optional if G 0:

- A x = mask missing: 0 = do not mask input missing on output; 1 = set missing input pixels to missing output pixels

Required if F 1:

B x = byte stretch if converting metric M to bytes.

For metrics bounded in [0.0, 1.0] only: (M in [2, 3, 6, 8, 10-15, 20-24, 31-38, 40, 42, 49])

1. From metric value in [0.0, 1.0] to byte in [0, 100]
2. From metric value in [0.0, 1.0] to byte in [0, 254]

For all metrics M except 1, 9, 16, 17, 18, 19, 25, 27:

3. From metric value in [Min, Max] to byte in [0, 254]
4. From metric value in [0.0, Max] to byte in [0, 254]
5. From metric value in [0.0, Max] to byte in [0, 100]

For metrics M in [1, 9, 16, 17, 18, 19, 25, 27] only:

6. No stretch allowed; the metric value is converted to byte

Required for metrics M: 21, 22, 23, 24:

X x = target code 1 (t1) (x in [0,100]).

Required for metrics M: 23, 24:

Y x = target code 2 (t2) (x in [0,100]).

Required for metric M 49:

K x = target difference level (k*) (x in [0, 100]).

4. *GraySpatCon* metrics

GraySpatCon notation is summarized in Table 1. The metrics computed by *GraySpatCon* (Table 2) use this notation to distinguish between "frequency" metrics and "adjacency" metrics. The frequency metrics are calculated based on the frequencies of individual pixel values. The adjacency metrics are calculated based on the adjacency matrix, which contains the frequencies of each type of adjacency (Fig. 3). Where appropriate, two versions of a given metric are provided for the two cases of "ordered" and "unordered" adjacencies (Fig. 3). All adjacency matrices are constructed by counting each adjacency once using a "2-neighbor" rule (yielding an ordered adjacency matrix); an unordered adjacency matrix is constructed by collapsing the ordered matrix across the main diagonal (Fig. 3).

Figure 3. Illustration of the ordered and unordered adjacency matrices for a 2-neighbor rule defined as "one pixel below and one pixel to the right." The pixel values labeled as 0, 1, 2, 3 may represent nominal or ordinal values. Adjacency frequencies and proportions are calculated as defined in Table 1. Adjacencies at the map border are ignored. The total number of adjacencies is 40, and the numbers of unique adjacencies in the ordered and unordered matrices are 16 and 10, respectively.

Input map with four pixel values, 25 pixels, and 40 adjacencies

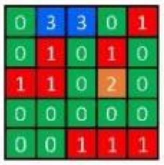

Adjacency matrices (ordered)

Frequency $x(i,j)$

	0	1	2	3
0	12	9	1	1
1	6	4	1	0
2	2	0	0	0
3	2	1	0	1

Proportion $p(i,j)$

	0	1	2	3
0	0.300	0.225	0.025	0.025
1	0.150	0.100	0.025	0
2	0.050	0	0	0
3	0.050	0.025	0	0.025

Adjacency matrices (unordered)

Frequency $x'(i,j)$

	0	1	2	3
0	12	15	3	3
1	-	4	1	1
2	-	-	0	0
3	-	-	-	1

Proportion $p'(i,j)$

	0	1	2	3
0	0.300	0.375	0.075	0.075
1	-	0.100	0.025	0.025
2	-	-	0	0
3	-	-	-	0.025

Table 1. Notation for metric computation.

Notation	Definition	Notes
Notation for metrics based on the frequency of pixel values (gray levels)		
i	Gray level of the frequency array	$i \in \begin{cases} (0, 100)\ if\ zero\ gray\ level\ is\ included \\ (1, 100)\ if\ zero\ gray\ level\ is\ excluded \end{cases}$
N_g	Number of gray levels	
$x(i)$	Element *i* in the frequency array	
$p(i)$	$\frac{x(i)}{\sum_i x(i)}$	
μ	$\sum_i [\, i \cdot p(i) \,]$	
σ^2	$\sum_i [\, (i-\mu)^2 \cdot p(i) \,]$	
t_1	User-selected gray level	Target code 1
Notation for metrics based on the frequency of pixel value (gray level) adjacencies		
$i\ and\ j$	Gray levels *i* (row) and *j* (column) in the adjacency matrix	$i, j \in \begin{cases} (0, 100)\ if\ zero\ gray\ level\ is\ included \\ (1, 100)\ if\ zero\ gray\ level\ is\ excluded \end{cases}$
N_g	Number of gray levels in the adjacency matrix	
R	Range of gray levels	
$x(i,j)$	Element *i, j* in the adjacency matrix	Ordered adjacencies
$x'(i,j)$	$\begin{cases} x(i,j) + x(j,i), if\ i \neq j \\ x(i,j), if\ i = j \end{cases}$	Unordered adjacencies
$p(i,j)$	$\frac{x(i,j)}{\sum_i \sum_j x(i,j)}$	
$p'(i,j)$	$\frac{x'(i,j)}{\sum_i \sum_j x(i,j)}$	
$p_x(i)$	$\sum_j p(i,j)$	
$p_y(j)$	$\sum_i p(i,j)$	
μ_x	$\sum_i [\, i \cdot p_x(i) \,]$	
μ_y	$\sum_j [\, j \cdot p_y(j) \,]$	

σ_x^2	$\sum_i [(i - \mu_x)^2 \cdot p_x(i)]$	
σ_y^2	$\sum_j [(j - \mu_y)^2 \cdot p_y(j)]$	
$p_{x-y}(k)$	$\sum_i \sum_{j \ where \ \lvert i-j \rvert = k} p(i,j)$	$k \in \begin{cases} (0, 100) \ if \ zero \ gray \ level \ is \ included \\ (0, 99) \ if \ zero \ gray \ level \ is \ excluded \end{cases}$
$p_{x+y}(k)$	$\sum_i \sum_{j \ where \ i+j = k} p(i,j)$	$k \in \begin{cases} (0, 200 \ ) \ if \ zero \ gray \ level \ is \ included \\ (2, 200) \ if \ zero \ gray \ level \ is \ excluded \end{cases}$
N_k	Number of *k* levels in the adjacency matrix	
t_1	User-selected gray level	Target code 1
t_2	User-selected gray level	Target code 2
k^*	User-selected *k* level	

Table 2. ***GraySpatCon*** **metrics. Columns 1-2: the metric number and short name as used in the parameter popup window (GTB) and the file gsc-parameters.txt (GWB). Column 3: metric description ("[MX]" in equation indicates substitution of metric number X; "[ref. X]" refers to literature cited number X). Column 4: Input data Type ("A" = all, "N" = nominal, "O" = ordinal). Column 5: Output data Type and [bounding range, if any] ("I" = integer, "N" = nominal, "R" = real).**

Metric (1)	Short name (2)	Description (3)	IT (4)	OT (5)
1	Mean	Mean: $\sum_i [\, i \cdot p(i) \,]$	O	R [0,100]
2	EvennessOrderedAdj	Evenness (ordered adjacencies) [ref. 7]: $\frac{[M4]}{2 \cdot \log N_g}, \; for\ N_g > 1$	A	R [0,1]
3	EvennessUnorderedAdj	Evenness (unordered adjacencies) [ref. 8]: $\frac{[M5]}{[\log(N_g^2+N_g) - \log 2]}, \; for\ N_g > 1$	A	R [0,1]
4	EntropyOrderedAdj	Entropy (ordered adjacencies) [ref. 1]: $-\sum_i \sum_j [\, p(i,j) \cdot \log p(i,j)], for\ p(i,j) > 0$	A	R
5	EntropyUnorderedAdj	Entropy (unordered adjacencies) [ref. 8]: $-\sum_i \sum_{j \geq i} [\, p'(i,j) \cdot \log p'(i,j)] \,, for\ p(i,j) > 0$	A	R
6	DiagonalContagion	Diagonal Contagion [ref. 10]: $\sum_i p(i,i)$	A	R [0,1]
7	ShannonDiversity	Shannon Diversity (gray levels): $-\sum_i [\, p(i) \cdot \log p(i) \,] \,, for\ p(i) > 0$	A	R
8	ShannonEvenness	Shannon Evenness (gray levels): $\frac{[M7]}{\log Ng}, \; for\ N_g > 1$	A	R [0,1]
9	Median	Median: $median\ [x(i)]$	O	I [0,100]
10	GSDiversity	Gini-Simpson Diversity (gray levels): $1 - \sum_i p(i)^2$	A	R [0,1]
11	GSEvenness	Gini-Simpson Evenness (gray levels): $\frac{[M10]}{1-(1/N_g)}, \; for\ N_g > 1$	A	R [0,1]
12	EquitabilityOrderedAdj	Equitability (ordered adjacencies) [ref. 6]: $\frac{[M14]}{1-(1/N_g^2)}, \; for\ N_g > 1$	A	R [0,1]
13	EquitabilityUnorderedAdj	Equitability (unordered adjacencies) [ref. 11]: $\frac{[M15]}{1-[\,2/(N_g^2+N_g)\,]}, \; for\ N_g > 1$	A	R [0,1]
14	DiversityOrderedAdj	Gini-Simpson Diversity (ordered adjacencies) [ref. 1, 6]: $1 - \sum_i \sum_j [\, p(i,j) \cdot p(i,j)]$	A	R [0,1]
15	DiversityUnorderedAdj	Gini-Simpson Diversity (unordered adjacencies): $1 - \sum_i \sum_{j \geq i} [\, p'(i,j) \cdot p'(i,j)]$	A	R [0,1]
16	Majority	Majority: $i \mid p(i) = maximum[p(i)]$	A	I [0,100]
17	LandscapeMosaic19	Landscape Mosaic(19 classes; see LM product sheet): $Ternary\ classification\ of\ [\, p(1), p(2), p\,(3)],\ iff\ \sum_{i=1}^{3} p(i) = 1$	N	N [1,19]

18	LandscapeMosaic103	Landscape Mosaic(103 classes; see LM product sheet): $Ternary\ classification\ of\ [\,p(1), p(2), p\ (3)],\ iff\ \sum_{i=1}^{3} p(i) = 1$	N	N [1,103]
19	NumberGrayLevels	Number of Gray Levels: N_g	A	I [1,100]
20	MaxAreaDensity	Maximum Area Density: $maximum[\ p(i)\ ]$	A	R [0,1]
21	FocalAreaDensity	Focal Area Density: $p(t_1)$	A	R [0,1]
22	FocalAdjT1	Focal adjacency (t_1): $\frac{\sum_j x'(t_1,j)}{\sum_i \sum_j x(i,j)}$	A	R [0,1]
23	FocalAdjT1andT2	Focal adjacency (t_1 and t_2) [ref. 15]: $\begin{cases} \frac{x(t_1,t_2)+x(t_2,t_1)}{\sum_i \sum_j x(i,j)}, if\ t_1 \neq t_2 \\ \frac{x(t_1,t_1)}{\sum_i \sum_j x(i,j)}, if\ t_1 = t_2 \end{cases}$	A	R [0,1]
24	FocalAdjT1givenT2	Focal adjacency (t_2 given t_1) [ref. 9, 14]: $\begin{cases} \frac{x(t_1,t_2)+x(t_2,t_1)}{\sum_j x'(t_1,j)}, if\ t_1 \neq t_2 \\ \frac{x(t_1,t_1)}{\sum_j x'(t_1,j)}, if\ t_1 = t_2 \end{cases}$	A	R [0,1]
25	StandardDeviation	Standard Deviation (population estimator): $\sqrt{\sum_i [\ (i-\mu)^2\ \cdot\ p(i)\ ]}$	O	R [0,100]
26	CoefficientVariation	Coefficient of Variation: $100 \cdot \left(\frac{[M25]}{\mu}\right), for\ \mu > 0$	O	R
27	Range	Range: $maximum(i) - minimum(i)$	O	I [0,100]
28	Dissimilarity	Dissimilarity] [ref. 2]: $\sum_i \sum_j [\ \lvert i-j \rvert\ \cdot p(i,j)$	O	R
29	Contrast	Contrast [ref. 1, 3]: $\sum_i \sum_j [\ (i-j)^2\ \cdot p(i,j)\ ]$	O	R
30	UniformityOrderedAdj	Uniformity (ordered adjacencies) [ref. 1]: $\sum_i \sum_j [\,p(i,j)\ \cdot\ p(i,j)]$	A	R [0,1]
31	UniformityUnorderedAdj	Uniformity (unordered adjacencies): $\sum_i \sum_{j \geq i} [\,p'(i,j)\ \cdot\ p'(i,j)]$	A	R [0,1]
32	Homogeneity	Homogeneity [ref. 1, 2]: $\sum_i \sum_j [\frac{p(i,j)}{1+(i-j)^2}]$	O	R [0,1]
33	InverseDifference	Inverse Difference [ref. 3]: $\sum_i \sum_j [\frac{p(i,j)}{1+\lvert i-j \rvert}]$	O	R [0,1]
34	SimilarityRMax	Similarity (R = 100) [ref. 5]: $1 - \sum_i \sum_j \left[\frac{\lvert i-j \rvert \cdot p(i,j)}{100}\right]$	O	R [0,1]
35	SimilarityRGlobal	Similarity (R = global range) [ref. 5]: $1 - \sum_i \sum_j \left[\frac{\lvert i-j \rvert \cdot p(i,j)}{global\ range}\right]$	O	R [0,1]
36	SimilarityRWindow	Similarity (R = window range) [ref. 5]: $1 - \sum_i \sum_j \left[\frac{\lvert i-j \rvert \cdot p(i,j)}{window\ range}\right]$	O	R [0,1]

37	DominanceOrderedAdj	Dominance (ordered adjacencies) [ref. 2]: $maximum[\, p(i,j)\,]$	A	R [0,1]
38	DominanceUnorderedAdj	Dominance (unordered adjacencies): $maximum[\, p'(i,j)\,]$	A	R [0,1]
39	DifferenceEntropy	Difference Entropy [ref. 1]: $-\sum_k [\, p_{x-y}(k) \cdot \log p_{x-y}(k)\,], for\ p_{x-y}(k) > 0$	O	R
40	DifferenceEvenness	Difference Evenness [ref. 11]: $\frac{[M39]}{\log N_k},\ for\ N_k > 1$	O	R [0,1]
41	SumEntropy	Sum Entropy [ref. 1]: $-\sum_k [\, p_{x+y}(k) \cdot \log p_{x+y}(k)], for\ p_{x+y}(k) > 0$	O	
42	SumEvenness	Sum Evenness [ref. 11]: $\frac{[M41]}{\log N_k},\ for\ N_k > 1$	O	R [0,1]
43	AutoCorrelation	Autocorrelation [ref. 2]: $\sum_i \sum_j [\, (i \cdot j) \cdot p(i,j)\,]$	O	R
44	Correlation	Correlation [ref. 1, 3]: $\sum_i \sum_j \left[\left(\frac{i-\mu_x}{\sigma_x}\right) \cdot \left(\frac{j-\mu_y}{\sigma_y}\right) \cdot p(i,j) \right], for\ \sigma_x, \sigma_y > 0$	O	R [-1,1]
45	ClusterShade	Cluster Shade [ref. 4]: $\sum_i \sum_j \left[\left(i + j - \mu_x - \mu_y\right)^3 \cdot p(i,j) \right]$	O	R
46	ClusterProminence	Cluster Prominence [ref. 4]: $\sum_i \sum_j \left[\left(i + j - \mu_x - \mu_y\right)^4 \cdot p(i,j) \right]$	O	R
47	RootMeanSquare	Root Mean Square: $\sqrt{\sum_i [\, i^2 \cdot p(i)\,]}$	O	R
48	AverageAbsDeviation	Average Absolute Deviation: $\sum_i [\, \lvert i - \mu \rvert \cdot p(i)\,]$	O	R
49	kContagion	k-contagion [ref. 11]: $\sum_{k=0}^{k^*} p_{x-y}(k)$	O	R [0,1]
50	Skewness	Skewness: $\frac{\sum_i [\, (i-\mu)^3 \cdot p(i)\,]}{\sigma^3}, for\ \sigma > 0$	O	R
51	Kurtosis	Kurtosis: $\frac{\sum_i [\, (i-\mu)^4 \cdot p(i)\,]}{\sigma^4}, for\ \sigma > 0$	X	R

5. Compiling and using *GraySpatCon* in standalone mode

This section is relevant ONLY if you want to use *GraySpatCon* in standalone mode and NOT its implementation in GTB or any other software mentioned in Section 3.

Compiling and environment

gcc -std=c99 -m64 -O2 -Wall -fopenmp grayspatcon.c -o GraySpatCon -lm

GraySpatCon recognizes an optional environment variable to specify the number of cores to use. If this is not specified, then *GraySpatCon* will use all cores of the operating system. Example for using N cores: **export OMP_NUM_THREADS=N**

Input files

The program looks in the current directory for:

Required: gscinput an 8-bit input file

Required: gscpars.txt - parameter file. In addition to the settings in gsc-parameters.txt (see Section 3) the following two parameters must be added to gscpars.txt:

R x = number of rows in input map (x is a positive integer)

C x = number of columns in input map (x is a positive integer)

Output files

For moving window analyses, the program writes the output map file gscoutput into the current directory. For global analyses, the program writes the output plain text file gscoutput.txt into the current directory.

File formats

Unlike the GTB and GWB implementations, which accept input as 8-bit (Geo)TIFF images and automatically convert output to 8-bit or 32-bit (Geo)TIFF images, running *GraySpatCon* in standalone mode supports file I/O *only* in the BSQ data format (sometimes referred to as ENVI format). The input data must be a flat, binary series of 8-bit values in the data range [0, 100] plus 255 indicating missing values. Importantly, *GraySpatCon* does not support the standard BSQ header files, and the R and C parameter values must be copied from the header file into the gscpars.txt parameter file for *GraySpatCon*.

To prepare an input BSQ, one option is gdal_translate, for example:

gdal_translate -of ENVI -ot Byte <filename>.tif gscinput.bsq

The extension ".bsq" must be removed before running *GraySpatCon*.

GraySpatCon writes BSQ flat binary files. To view the output BSQ file in an image viewer or a GIS, it is necessary to construct an appropriate header file for the (renamed) gscoutput.bsq image. This can usually be done by editing a copy of the original input header file. This can be as simple as changing the "data type" in an ENVI-style header (from "1" to "4" for float output). For ESRI BSQ headers attention must be given to the data type field (e.g., unsigned int versus float), the missing value or NODATA field (see missing data codes in Section 2.1), specifying the number of bits per pixel (8 or 32), number of bytes in a band and row (same as input if the output is byte, or 4*input if output is float), etc. If needed for ESRI BSQ files, the original projection file (gscinput.prj) can be copied to the output (gscoutput.prj). For ENVI files the projection should be contained within the header file. The header file and projection information are required when converting output to a (Geo)TIFF image via gdal_translate.

The above suggestions do not cover the range of format or software possibilities but should be enough to guide a knowledgeable user.

6. Literature cited

(1) Haralick RM, Shanmugam K, Dinstein I. 1973. Textural Features for Image Classification. In: IEEE Transactions on Systems, Man, and Cybernetics, vol. SMC-3, no. 6, pp. 610-621. https://doi.org/10.1109/TSMC.1973.4309314

(2) Soh L-K, Tsatsoulis C. 1999. Texture analysis of SAR sea ice imagery using gray level co-occurrence matrices. IEEE Transactions on Geoscience and Remote Sensing 37:780-795. https://doi.org/10.1109/36.752194.

(3) Clausi, DA. 2002. An analysis of co-occurrence texture statistics as a function of grey level quantization. Canadian Journal of Remote Sensing 28:45-62. https://doi.org/10.5589/m02-004

(4) Conners RW, Trivedi MM, Harlow CA, 1984. Segmentation of a high-resolution urban scene using texture operators. Comput. Vision, Graph., Image Processing, vol. 25, pp. 273–310.

(5) Gower JC. 1971. A general coefficient of similarity and some of its properties. Biometrics 27: 857–871. https://doi.org/10.2307/2528823

(6) Wickham JD, Riitters KH. 1995. Sensitivity of landscape metrics to pixel size, International Journal of Remote Sensing 16:3585-3594. https://doi.org/10.1080/01431169508954647

(7) Li H, Reynolds JF. 1993. A new contagion index to quantify spatial patterns of landscapes. Landscape Ecology 8:155–162. https://doi.org/10.1007/BF00125347

(8) Riitters KH, O'Neill RV, Wickham JD, Jones KB. 1996. A note on contagion indices for landscape analysis. Landscape Ecology 11:197–202. https://doi.org/10.1007/BF02071810

(9) Riitters K, Wickham J, O'Neill R, Jones B, Smith E. 2000. Global-scale patterns of forest fragmentation. Conservation Ecology 4(2): 3. http://www.consecol.org/vol4/iss2/art3/

(10) Riitters KH, O'Neill RV, Hunsaker CT, et al. 1995. A factor analysis of landscape pattern and structure metrics. Landscape Ecology 10:23–39. https://doi.org/10.1007/BF00158551

(11) Riitters K, Vogt P. Submitted. GraySpatCon: moving window analysis of landscape patterns with frequency- and adjacency-based metrics.

(12) Vogt P, Riitters K. (2017). GuidosToolbox: universal digital image object analysis. European Journal of Remote Sensing 50:352-361. https://doi.org/10.1080/22797254.2017.1330650

(13) Vogt P, Riitters K, Rambaud P, d'Annunzio R, Lindquist E, Pekkarinnen A. (2022). GuidosToolbox Workbench: spatial analysis of raster maps for ecological applications, Ecography 2022: e05864. https://doi.org/10.1111/ecog.05864

(14) Wade T G, Riitters KH, Wickham JD, Jones KB. 2003. Distribution and causes of global forest fragmentation. Conservation Ecology 7(2). http://www.consecol.org/vol7/iss2/art7

(15) Riitters KH, O'Neill RV, Jones KB. 1997. Assessing habitat suitability at multiple scales: A landscape-level approach. Biological Conservation 81:191-202. https://doi.org/10.1016/S0006-3207(96)00145-0
