# Supplementary figures and images for: Mapping landscape ecological patterns using numeric and categorical maps
Source: PLoS One. 2023 Nov 15;18(11):e0291697. doi: 10.1371/journal.pone.0291697 (PMC10651036; doi:10.1371/journal.pone.0291697)

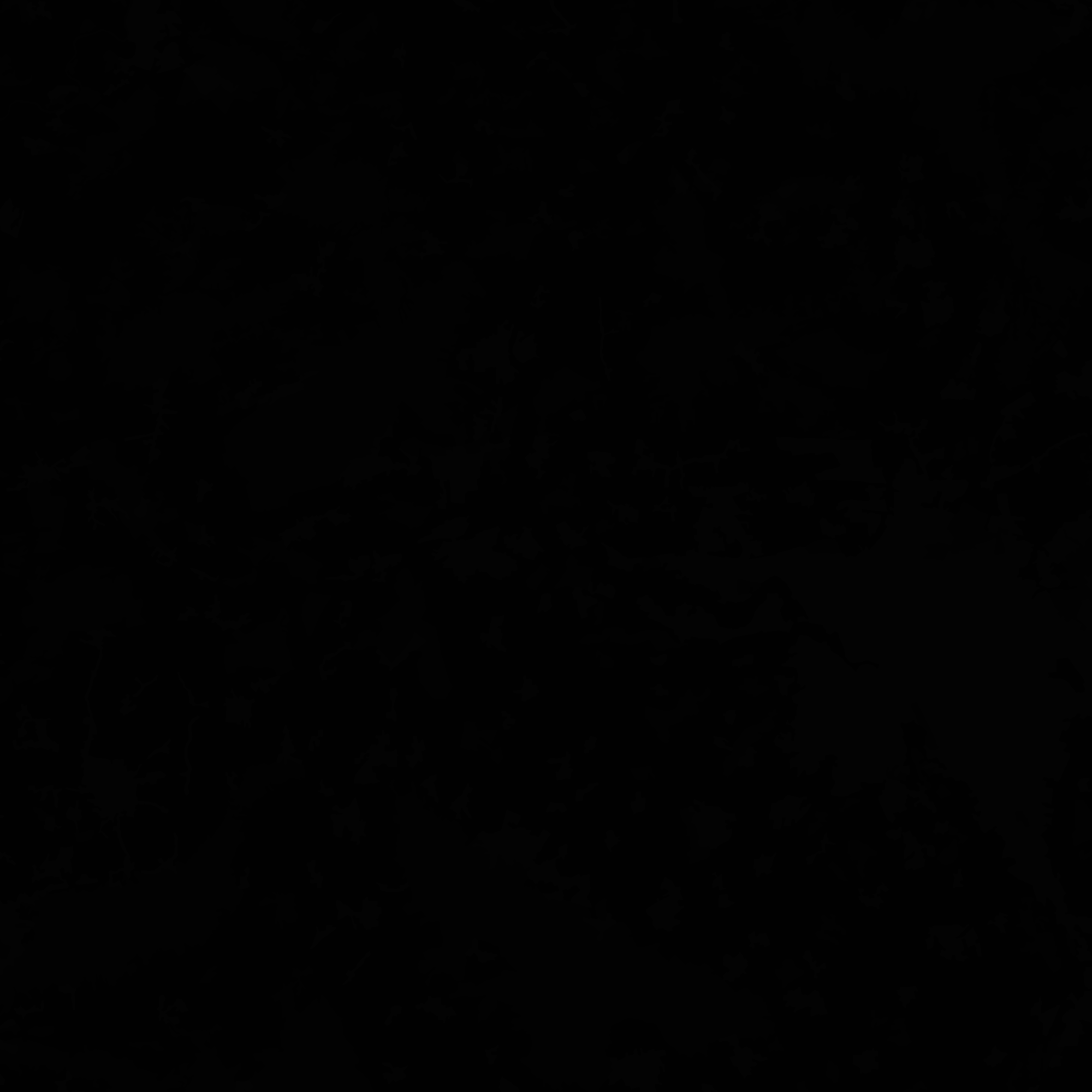

Supplement: S2 File — (ZIP) [file pone.0291697.s002.zip › S2_Example_Data_Rscripts/clc3class.tif]

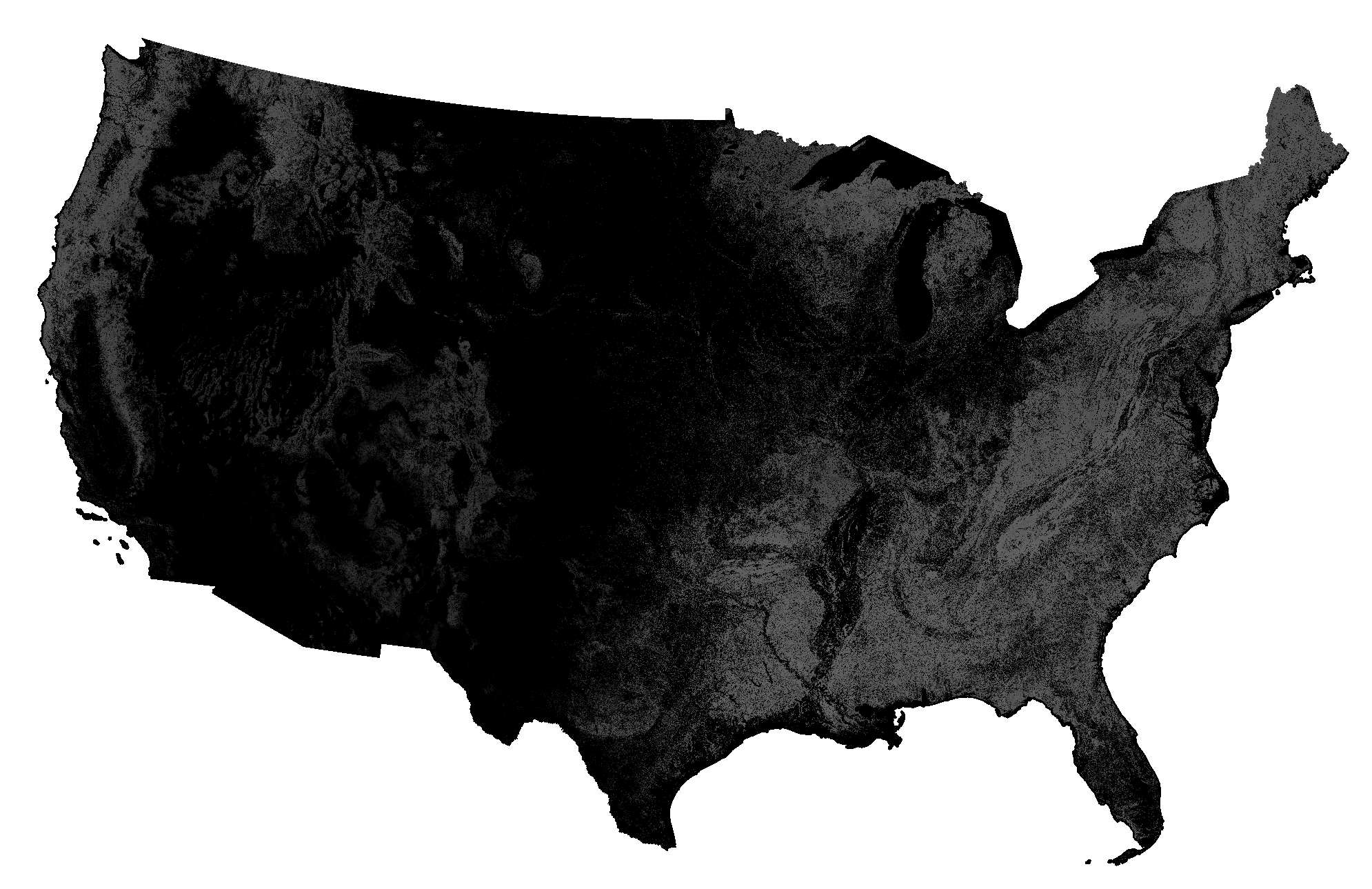

Supplement: S2 File — (ZIP) [file pone.0291697.s002.zip › S2_Example_Data_Rscripts/gscinput.tif]
